# Effect of Polishing Nozzle Wear Evolution on BK7 Topography

**DOI:** 10.3390/ma18081796

**Published:** 2025-04-14

**Authors:** Xuhong Chen, Haihong Pan, Lin Chen, Hui You, Xubin Liang

**Affiliations:** 1 School of Advanced Manufacturing Engineering, Guangxi Science and Technology Normal University, Laibin 546100, China; 18172423561@163.com; 2 Precision Polishing and Measuring Laboratory, Guangxi University, Nanning 530000, China; hustphh@163.com (H.P.); gxdxcl@163.com (L.C.); gxnnlxbwlh@163.com (X.L.)

**Keywords:** abrasive water jet (AWJ), stainless steel 304 coatings, nozzle wear, simulation

## Abstract

In ultra-precision polishing tests, due to the corrosive and adhesive properties of the polishing abrasive, the spray system faces wear, blockage and oxidation problems. To solve these problems, this paper studied nozzles and verified the wear mechanism of the coated and uncoated nozzles by simulating the operating conditions after assembling the spray system. In the early stages of the experiment, the polishing speed of the spray system (*v* = 10 L/min), the feed rate (*v*_f_ = 7.8 mm/min) and the polishing pressure (2~3.5 MPa) were maintained. The wear mechanism and surface morphology features of the nozzles in each case were analyzed by Hitachi S-3400N electron microscopy. When comparing the surface morphology of the nozzle coated with titanium alloy and the uncoated one, the results show that there is a significant difference in the corrosion resistance of the coatings to the abrasive particles. A significant effect was seen on the wear morphology, proving that the nozzle wear mechanism includes wear, adhesion and diffusion. Under the experimental conditions of a lateral velocity of 7.8 mm/min and a polishing force of 2 MPa, BK7 was polished using nozzles 1 and 2, resulting in a surface roughness of 75 nm and 35 nm while PV values were 125 nm and 67 nm, respectively. The excellent quality of nozzle 2 (coating nozzle) was proven, further demonstrating the superiority of the coating nozzle. Finally, the lifespan of the nozzle was extended and the surface accuracy of BK7 was improved by coating titanium alloy composite material on the 304 stainless steel nozzle.

## 1. Introduction

**Figure 1:**
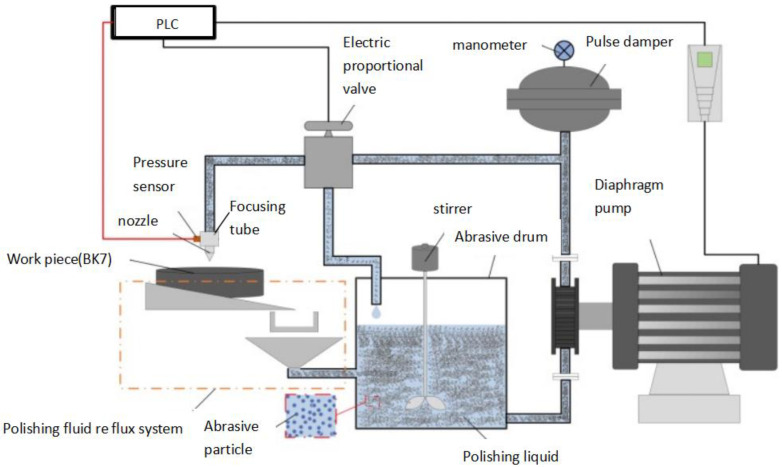
Principle of abrasive water jet process.

The ultra-precision polishing system is a non-traditional polishing equipment that use low-pressure (2–3.5 MPa) water to accelerate abrasive particles (grid # 80) to erode the target material. The material 304 stainless steel is widely used in the manufacturing field, especially suitable for nozzle raw materials in spray devices. Stainless steel is well known for its corrosion resistance, high durability, and esthetics, and steel exhibits a high degree of plasticity. Compared with ferritic stainless steel and martensitic stainless steel, 304 stainless steel (austenite) has a higher work hardening rate, and there are significant differences in mechanical strength and physical properties including elongation and thermal conductivity [1]. It is considered that higher hardness and mechanical strength will cause stainless steel workpieces to wear out quickly and increase the cutting zone temperature, thereby affecting the surface topography of the polishing parts [2,3,4]. Since strong oxidizing agents such as cerium oxide and aluminum oxide are often used in ultra-precision polishing, this study primarily evaluates whether the surface wear of the 304 stainless steel nozzle is suitable for ultra-precision polishing. We usually use surface coatings to resist corrosion on stainless steel surfaces. Wagner et al. studied and defined tool wear during the milling process of the Ti-1023 alloy, helping tool manufacturers and users extend tool life [5]. Ginting et al. found that there was no wear and brittle fracture (cracking, peeling, and forming) in the grinding process of titanium alloys, thereby improving the surface finishing speed [6]. Rahman et al. used cutting tools to perform dry high-speed machining of stainless steel 304 and used a newly developed internal PVD coating to solve the problem of tool wear [7]. Chen et al. described the detailed composition of tools and their differences and microstructure [8]. Suarez et al. studied the changes in wear patterns under a high-pressure coolant at 80 bar and a rotational speed of 30 m/min in 2017 [9]. They found that the wear pattern of the nozzle changed from lateral wear (reduced by 30%) to notch wear, and the cutting force was reduced by 10%. Cavitation is a very important material removal method in ultra-precision polishing. The cavitation jet produces a cavitation cloud, which will cause serious damage to hydraulic equipment and jet system [10,11]. The abrasive particles can cause deep wear on the metal surface, leading to pits and cracks [12]. Through the analysis and comparison of different particle sizes and densities, Narayanaswamy et al. studied the removal rate of affected materials by SEM and found the removal efficiency of alumina abrasive particles was higher than that of silicon oxide [13]. Fernandez et al. found that AlTiSiN and AlTiN are the best coatings with crystalline structures, which can promote the formation of protective layers and slow down the evolution of coating wear on the cutting surface [14]. There is pitting wear and micro cutting in the processing of stainless steel 304, which forms severe notch grooves [15]. Due to the rapid development of coating technology, coating tools have been used as the main polishing tools for material removal in recent years [16,17,18]. Chen et al. found the TiAIN film coating on the nozzle remained stable [19]. Varadaraajan et al. analyzed the tool life and wear during physical vapor deposition and dual coating tools in five processing environments [20]. Fernandes et al. found the Tinal coating and Alcrn+ coating on tools improved the material removal rate significantly [21]. By analyzing and comparing the atomization parameters of different nozzles, Li et al. found that the PM value of the atomization method with added voltage is significantly suppressed [22]. Sidambe et al. provided an overview of the widespread applications of titanium and titanium alloys in the industrial and medical sectors [23]. Zhu et al. studied the consequences of the nozzle coating detachment and changes in water jet coating removal in the aerospace field [24]. Wang et al. studied the effect of key factors for polishing parameters on coating removal efficiency in abrasive jet polishing [25]. Liu et al. analyzed the experimental results of nozzle removal of workpiece coatings with different structures [26]. Song et al. studied the coating process of the top jet fluidized bed coating machine [27]. The variability of particle coatings can be evaluated through SEM measurement. Slavekina et al. summarized the rules of thermoplastic composite materials on the surface of coated nozzles and the composite process during nozzle manufacturing [28]. Koller et al. found that graphite and silicon carbide are effective to reduce nozzle wear by 7.5 times [29]. So it was concluded 15% volume silicon carbide should be contained in composite materials of the nozzle. The nozzle used for additive manufacturing of aviation parts was scanned through three different methods. Abruzzo et al. determined the correlation between surface texture and nozzle fluid dynamics [30]. Bierschenk et al. improved the nozzle structure to alleviate the agglomeration problem of abrasives, thereby making the thin film and coating on the sample more regular [31]. Wang et al. studied the influence of jet process parameters on the surface quality and cutting thickness of nozzle coatings [32]. Through single-factor experiments and analyzing the working results, the optimal surface roughness of the workpiece was obtained. Zheng et al. analyzed the erosion evolution process of multiple abrasive hard brittle and soft plastic materials, and more intuitively revealed the formation mechanism of brittle fracture and the plastic shear law [33]. At the same time, the mechanism of abrasive erosion plastic extrusion was proposed and the mechanism of jet polishing was improved. According to the pressure distribution and transverse shear force distribution curve of the workpiece surface, the pressure and transverse shear force on the workpiece will increase with the increase in the nozzle outlet wear. Chen et al. summarized that the diameter of the nozzle outlet was worn to 1.6 mm, the jet force diverged, and the workpiece was seriously damaged [34]. The increase in injection pressure and transverse shear force affects the machining quality of the workpiece surface. It eventually leads to a reduction in the surface roughness and surface finish of the workpiece (BK7). Through the analysis of variance, it was found that the surface roughness of the nozzle affects the surface accuracy of BK7 and as the surface roughness of the nozzle increases, the surface accuracy of BK7 decreases. The life cycle of coated and uncoated nozzles was studied in terms of the surface accuracy required for BK7 processing and the principle of the test is shown in Figure 1. The experiments used the surface precision (30 nm) of BK7 as the standard.

## 2. Methodology

### 2.1. Materials and Tools (Nozzle Design and Fabrication)

**Figure 2:**
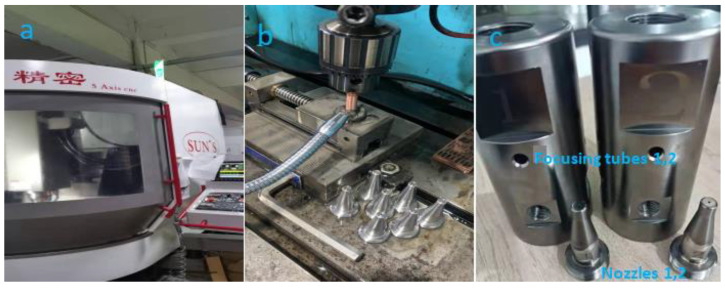
Fabrication process for nozzles and focusing tube. (**a**) Machining nozzle and focus tube on CNC SUN’5 Axis lathe, (**b**) clamp of nozzle cavity, (**c**) experimental sample of nozzle and focusing tube.

**Figure 3:**
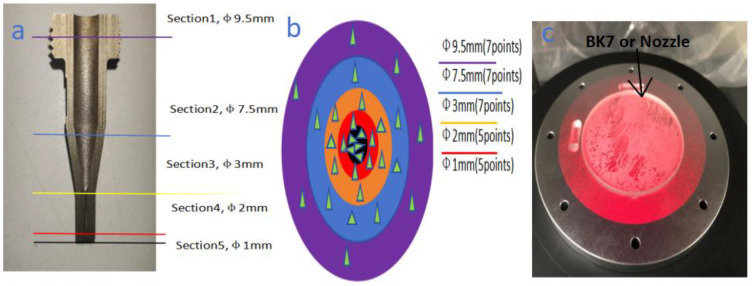
Cross-section roughness test of nozzle 1 and nozzle 2. (**a**) Cross-section of nozzles, (**b**) number of pickup points per section of nozzles, (**c**) ZYGO laser interferometer platform.

Machining tests were carried out on CNC 5 Axis lathe (SUN, San Diego, CA, USA), which is shown in Figure 2a. The nozzle material is 304 stainless steel, and it has a hardness of 200 Hv. The mechanical and physical properties and chemical composition of the nozzles are shown in Table 1. The 304 stainless steel is widely used in the production of equipment and parts requiring good comprehensive properties which include corrosion resistance and form ability [35]. In order to maintain the inherent corrosion resistance of stainless steel, the steel must contain more than 18% chromium, more than 8% nickel content and needs to be coated. Uncoated metal nozzle tools (Figure 1) and coated nozzles were obtained from the same supplier. In the manufacturing process of the nozzle, the nozzle fixture and the focusing tube fixture need to be manufactured to keep the surface accuracy stable in the respective manufacturing process due to the high surface roughness requirement of the nozzle cavity. The fixture manufacture is shown in Figure 2b. After the turning of the respective fixture is completed and assembled on the CNC, the focusing tube and nozzle need to be manufactured on the CNC lathe shown in Figure 2a. The produced focusing tubes and nozzles are shown in Figure 2c. In order to be able to study the surface roughness of the nozzle cavities uniformly, it is necessary to do a destructive test on nozzle 1 and nozzle 2 before conducting the fatigue test and the locations for detecting the roughness are shown in Figure 3a. In the paper, the RMS of the nozzle cavity is detected in five areas and the quantity of every section is shown in Figure 3b. The number of key points detected in the five regions are different. This allows for selecting more detection points based on the axial mutation area of the nozzle flow channel, so that the resulting data are more in line with reality. It is shown in Figure 3b that a total of 31 key points were selected for the 5 regions.

**Figure 4:**
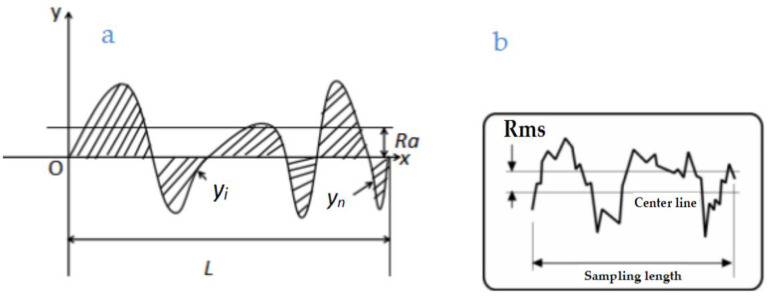
(**a**) Definition of Ra; (**b**) Definition of Rms.

The roughness of the key points in every section which includes nozzle 1 and nozzle 2 are shown in Table 2. All of the key points were detected by laser interferometer (ZYGO, Fremont, CA, USA), which is shown in Figure 4. The arithmetic average roughness (Ra) of the focusing tube should be less than that required in the figure. Due to the direct connection between the focusing tube and the nozzle thread, if the internal cavity size of the focusing tube is too rough, it can cause vortex turbulence and noise problems, which further causes the abrasive liquid flowing through the nozzle to diverge and further aggravates the nozzle wear. Thus, roughness restrictions were imposed on the focusing tube (see Table 3 for specific details).

According to the EN ISO 4287 [36], the parameter Ra or arithmetic mean deviation of the assessed profile, which is shown in Formula (1), is indeed one of several parameters used to quantify surface roughness. Ra, which is the root mean square (RMS) roughness and shown in Formula (2), provides a measure of the surface texture’s deviation from the mean line. Each parameter has its specific application and relevance, and understanding these differences is crucial for selecting the right measurement for a given application. In this paper, Ra and RMS were detected in a manner consistent with the EN ISO 4287. The definitions of Ra and RMS are shown in Figure 4a and Figure 4b, respectively.

(1) $$R_{a}=\int_{0}^{l}\left|y\right|d_{x}$$

(2) $$RMS=\sqrt{\frac{y_{1}^{2}+y_{2}^{2}+y_{3}^{2}+\ldots +y_{n}^{2}}{N}}$$

### 2.2. Simulation Results of Nozzle Cavity Erosion

**Figure 5:**
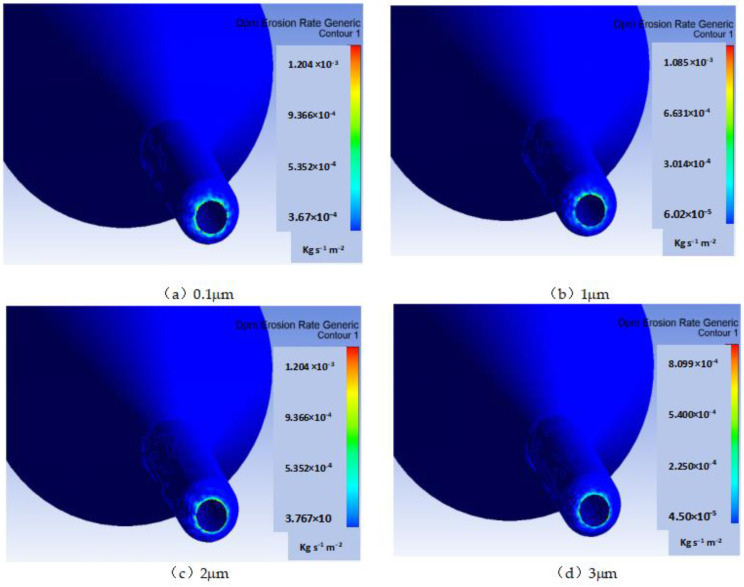
Erosion cloud image of nozzle cavity under different abrasive diameters.

**Figure 6:**
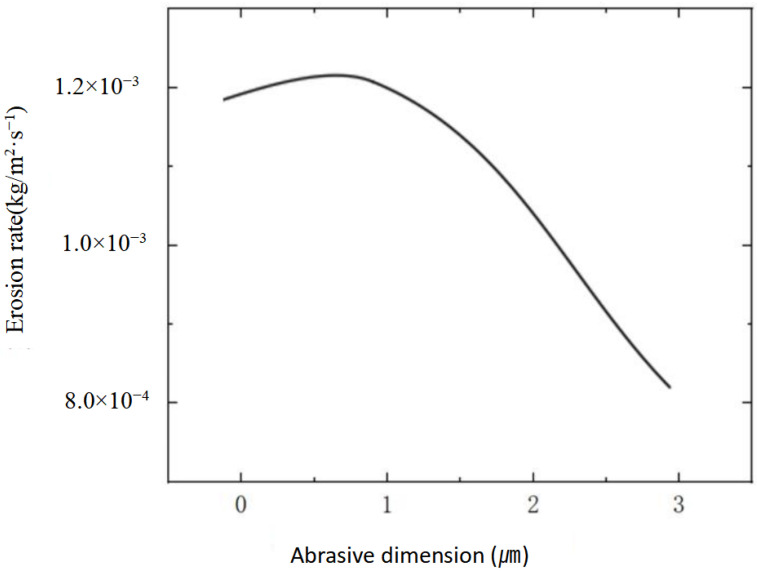
Variation from erosion rate of nozzle with different abrasive diameters.

In ultra-precision polishing, the wear degree of the nozzle outlet seriously affects the surface accuracy of polishing BK7. In order to ensure the corrosion resistance and wear resistance of the nozzle in the injection system, increasing the hardness of the nozzle and improving the accuracy of the workpiece become the priority. Although 304 stainless steel has the ability to wear and resist corrosion, the abrasive cerium oxide and alumina in the polishing jet are extremely corrosive. The 304 stainless steel will chemically react with the nozzle, resulting in corrosion and rust of the jet device in the jet system, especially the position of the nozzle outlet. Figure 5 shows the wear cloud diagram of nozzle cavity under four different abrasive diameters obtained by numerical simulation. The wear near the nozzle is mainly caused by the impact force of abrasive particles and chemical corrosion. As can be seen from the curve in Figure 6, the erosion rate of the particles in the nozzle cavity decreases with the increase in particle diameter in the range of 0.5 μm to 3 μm.

### 2.3. Polishing Performance Evaluation Experiment of Nozzles

**Figure 7:**
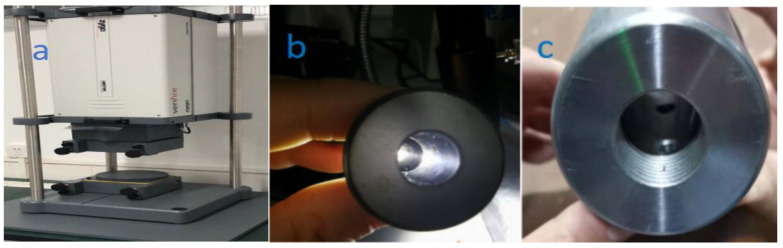
Detection of the nozzle and focusing tube (**a**) RMS (ZYGO, USA); (**b**) experimental sample of nozzle; (**c**) experimental sample of focusing tube.

The experiment verifies the corrosion resistance of the two nozzles on the ultra-precision polishing platform. The schematic diagram of the precision platform is shown in Figure 1. Before performing the experiment, the surface accuracy of all nozzles and focusing tubes are guaranteed almost as well. The RMS of the nozzles and focusing tube are detected by the ZYGO in Figure 7a and flash tester in Figure 7b,c.

The experiment was conducted in two stages with two sizes of abrasives, with 1 μm alumina in the first stage and 500 nm alumina in the second stage. All parameters of nozzle life test are shown in Table 4.

**Figure 8:**
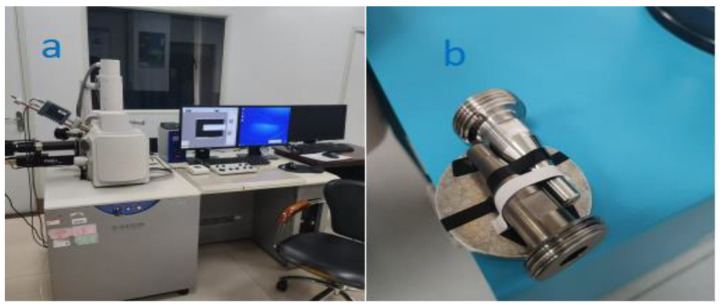
Detection of the nozzle (**a**) Hitachi S-3400N electron microscopy, (**b**) experimental sample of nozzles.

The same type of uncoated nozzle and nozzle coated with 0.02 mm thick titanium alloy were installed on the same assemble injection in Figure 1. The coatings of the nozzles are unique and are only suitable for the abrasive water jet system. The experiments were carried out successively on the ultra-precision polishing platform. In order to ensure the same operating conditions of the two nozzles in line with the actual ultra-precision operating conditions, the injection pressure was maintained at 2.0 MPa and 3.5 MPa during the experiment. The polishing platform runs for 8 h per day. After 60 days of experiments, the wear of the uncoated nozzles and titanium-coated nozzles was observed. The surface topography of the nozzle was obtained by Hitachi S-3400N electron microscopy (Hitachi Ltd., Tokyo, Japan) in Figure 8a. The nozzle one and nozzle two were detected, respectively, by the S-3400N electron microscopy in Figure 8b. Before detection, the nozzles should be cleaned carefully. A material deposition on the inner wall of the nozzle can easily cause blockage. By optimizing the amount of abrasive in the spraying medium, the material on the hole wall can be removed [37]. This removal method may be reasonable in some cases, but it is not suitable for the purpose of this article. It is supposed to achieve the surface smoothness of BK7 at the nanoscale, and a certain concentration of medium is essential to improve the nozzle’s wear resistance and anticlogging ability.

## 3. Results and Discussion

### 3.1. Surface Topography of Nozzles

**Figure 9:**
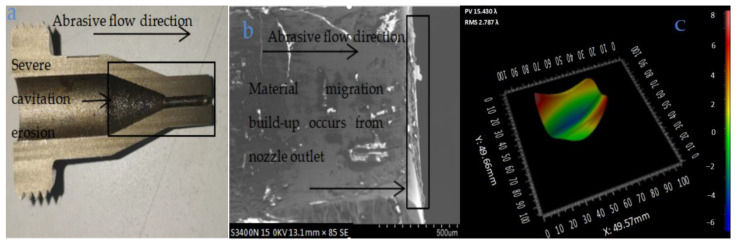
The 304 stainless steel nozzle 1: (**a**) nozzle profile; (**b**) partial enlarged view of nozzle 1 outlet; (**c**) nozzle outlet location topography.

To maintain the RMS of BK7 at 30 nm, the working limit time of the coated nozzle was within 480 h. Therefore, wear analysis of nozzle during 60 days of operation should be performed on the ultra-precision platform. The wear condition of 304 stainless steel nozzle should be tested after the fatigue test. The surface accuracy of BK7 processed by the uncoated nozzle was measured continuously during the 480 h test and it was found that the limit processing time of BK7 maintaining a surface roughness of 30 nm was 280 h. Figure 9 shows the wear condition of the surface of the 304 stainless steel with the uncoated nozzle after the test. It is shown in Figure 9a that the nozzle cavity has severe cavitation erosion. Due to the fineness of the abrasives and their adhesion to each other, it is difficult for 304 stainless steel to isolate the polishing powder. Because of the lower pressure (2–3.5 MPa) and the larger abrasive liquid concentration, the nozzle cavity is blocked easily. Figure 9b shows material migration and build-up occurs from the nozzle outlet of 304 stainless steel. It is also shown in Figure 9b that deformation of 304 stainless steel occurs in uncoated nozzles, and the 304 stainless steel does not have sufficient hardness to resist the abrasive flow impact. Figure 9c is the graphic of the laser interferometer measurement (ZYGO, USA) of the wear at the nozzle outlet position, belonging to detection of the boxed area shown in Figure 9b.

**Figure 10:**
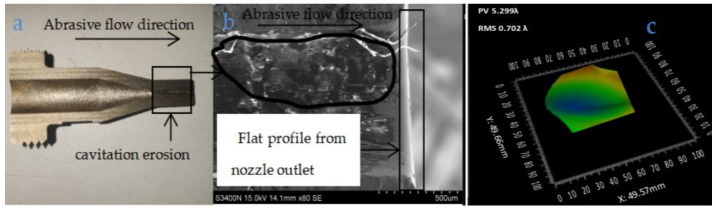
Composite titanium alloy of nozzle 2: (**a**) nozzle profile, (**b**) partial enlarged view of nozzle 2 outlet, (**c**) nozzle outlet location topography.

**Figure 11:**
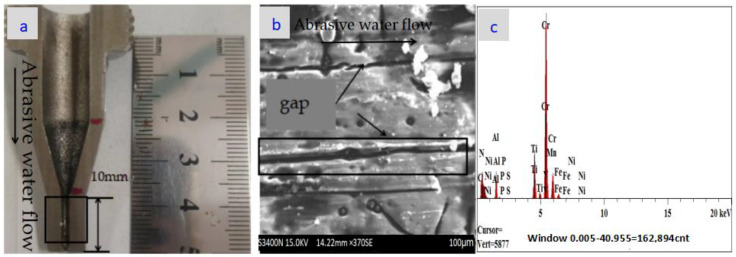
SEM images of nozzle 1 without coating after 480 h tests. (**a**) Experimental nozzle profile; (**b**) the wear area at the nozzle 1 exit position magnification, magnification 300×; (**c**) the main metal contents of the nozzle 1 outlet wear area.

**Figure 12:**
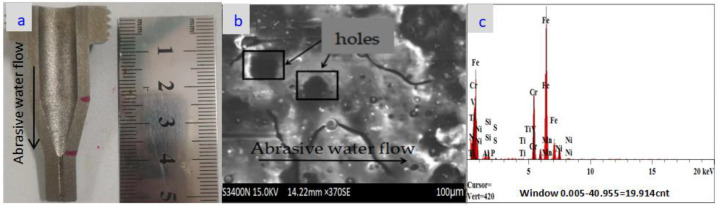
SEM images of nozzle 2 coated with titanium alloy composite after 480 h tests. (**a**) Experimental nozzle profile; (**b**) the wear area at the nozzle 2 exit position, magnification 300×; (**c**) the main metal contents of the nozzle 2 outlet wear area.

The nozzle cavity shown in Figure 10a shows a cavitation problem near the nozzle outlet position, which may be due to the lack of wear on the nozzle orifice, which resulted in a large amount of abrasive build-up in the nozzle cavity over a long period of time, causing cavitation erosion of the nozzle cavity. However, due to the reason that the nozzle orifice is plated, there is almost no wear problem, and the details are shown in Figure 10b. In optical component manufacturing and testing, PV is often used to describe the size of the overall bulge or depression on the surface of the optical component and to characterize the overall flatness of the surface. Figure 10c shows the RMS value and PV value after wear experiments at the nozzle outlet location of the maximum wear position. In Figure 10c, it can be seen that the nozzle’s PV is 5.299λ (for the wavelength of the ZYGO laser detector, λ has the value of 633 nm). Consider the nozzle wear problem from the nozzle inlet to the nozzle outlet which is shown in Figure 11a. Since the inlet and outlet of the nozzle should be at the position of the sudden change in pipe diameter, compared with the wear of the inner cavity of the grinder, the wear of the inlet and outlet of the nozzle is the most serious; this is verified after studying the wear of the entire length of the nozzle. The surface morphology of the nozzles was examined by a Hitachi S-3400N electron microscope which is shown in Figure 8a. According to the polishing experimental results in Figure 11b and Figure 12b, the surface wear problem of nozzle 1 is significantly higher than that of nozzle 2. It seems reasonable that nozzle 2 has a higher wear resistance than nozzle 1 from Figure 11c and Figure 12c, because the containment of Fe, C, and titanium in the nozzles are different. In the magnified wear images of nozzle in Figure 11b, large gaps and cracks can be observed at the cavity surface of nozzle 1. In Figure 11b and Figure 12b, there are some small holes and cracks marked in the wire frame; these were tested for their elemental contents, and the testing results are presented in Table 5 and Figure 11c and Figure 12c. The schematic diagrams of the metal content in the wear zone (marked in frame in Figure 11b and Figure 12b) of nozzles (1, 2) are shown in Figure 11c and Figure 12c, respectively. Table 5 lists the percentage of metal content in the wear zone at the outlets of nozzles 1 and 2 while Table 6 lists RMS values (before and after polishing) of two different nozzles (the nozzle 1 and nozzle 2).

**Figure 13:**
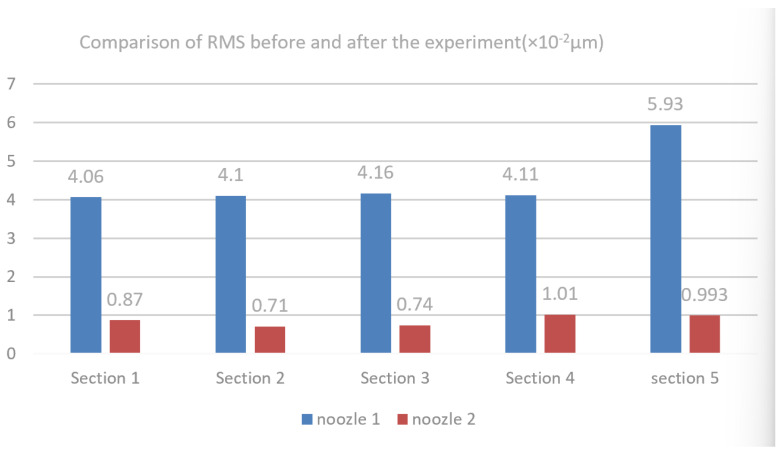
Comparison of RMS of nozzle 1 and nozzle 2.

Combined with the RMS of the five sections shown in Figure 4, the variation values of nozzle 1 and nozzle 2 in each section are obtained. It can be seen that nozzle 1 has the largest change (Section 5) in RMS, with a RMS change rate 30% to 44% higher than the other four areas in nozzle 1 and a roughness rate 6 times higher than the same area of the nozzle 2 shown in Figure 13.

### 3.2. Surface Topography of BK7

**Figure 14:**
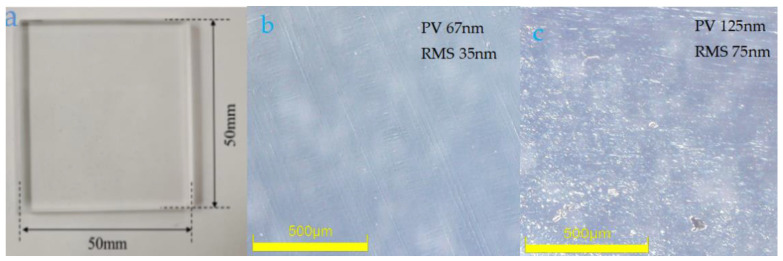
(**a**) Optical quality of BK7 after polishing, (**b**) the surface topography of BK7 with nozzle 2, (**c**) the surface topography of BK7 with nozzle 1.

The experimental parameters were obtained through variance analysis: the transverse speed was 7.8 mm/min; the mass flow rate of the mixture was 10 L/min. The abrasive particle size was 500 nm, and the hydraulic pressure was 2 MPa. BK7 was polished using nozzle 1 and nozzle 2, which were tested for durability on ultra-precision platforms. Firstly, the polishing parameters of the two nozzles were set to optimal parameters as mentioned above. The surface topography of BK7 was then observed with ultra-deep microscopy and ZYGO. The results are shown in Figure 13. The polished BK7 was a 50 × 50 cube with a thickness of 5 mm (Figure 14a). It is not difficult to see from Figure 14c that there are many processed spots and scratches on the surface of BK7, which are strictly prohibited in practical applications. The surface roughness of BK7 is 75 nm, and the PV value is 125 nm. As can be seen from Figure 14b, the processed surface of BK7 is uniform without obvious scratches and spots. The surface roughness of BK7 is 35 nm, and the surface topography is 67 nm.

## 4. Conclusions

The article analyzes the experimental results of using different nozzles to remove BK7 surface materials. The novelty of this article lies in the analysis of the different degrees of material erosion after durability testing of two different nozzles on an ultra-precision polishing platform. To maintain the surface roughness of BK7 at 30 nm, the working limit time of the coated nozzle is 480 h and the working limit time of the uncoated nozzle is 280 h or so, which is almost twice the former. The details are as follows:

(1) The nozzle 1 has the largest change in roughness, with a roughness change rate 30% to 44% higher than the other three areas and a roughness rate 6 times higher than the same area of the nozzle 2 after the 480 h test.
(2) The price of each stainless steel nozzle is 450 RMB, while the price of each coated nozzle is 500 RMB according to the supplier’s quotation. In addition, there are labor costs, processing costs, warehouse costs, and so on. From an economic perspective, improving the wear resistance of nozzles means extending their lifespan.
(3) Under the test conditions of transverse speed of 7.8 mm/min and polishing force of 2 MPa, nozzles 1 and 2 were used for polishing. The surface roughness of BK7 was 75 nm and 35 nm, while the PV values were 125 nm and 67 nm, respectively. The excellent quality of nozzle 2 (coated nozzle) is fully proven.
(4) As one of the key components of the injection system, the nozzle’s working quality seriously affects the performance of the injection system. Nozzle blockage seriously affects the operation of the precision polishing experiment. The reason for the blockage is the polishing medium on the nozzle or impurities in the circulating medium blocking the nozzle path, which causes the blockage of the entire injection system, which then causes irreversible damage to the entire ultra-precision platform.

## Figures and Tables

**Table 1:** Chemical composition and mechanical properties and physical properties of 304 stainless steel.

Chemical Composition (%)								
C	Si	Mn	P	S	CR	NI	N	Balance
≤0.08	0.75	2.0 MAX	0.045	0.03	≥18	≥8	0.10	Fe
**Mechanical Properties**								
**Yield Strength (MPa)**	**Tensile Strength (MPa)**		**Elongation**		**Shrink Percentage**		**Elasticity Modulus**	**Poisson’s Ratio**
≥205	≥520		δ5 (%) ≥ 40		ψ (%) ≥ 60		≤200	0.247
**Physical Property**								
**Density**	**Specific Heat Capacity**		**Thermal Conductivity**				**Coefficient of Linear Expansion**	
7.93 g/cm^3^	0.50 kJ·kg^−1^·K^−1^		16.3 W·m^−1^·K^−1^				17.2 × 10^−6^·K^−1^	

**Table 2:** RMS of different nozzles’ cavity before polishing.

Inspected Items	RMS Before Polishing (nm)	
	Nozzle Cavity (1)	Nozzle Cavity (2)
Ø 9.5 (mm) hole (Section 1)	107	146
	102	95
	104	97
	89	106
	99	98
	95	96
	93	98
Ø 6 (mm) hole (Section 2)	95	97
	74	91
	77	99
	91	91
	94	96
	99	91
	84	93
Ø 3 (mm) hole (Section 3)	97	95
	76	92
	75	97
	93	94
	92	93
	96	94
	83	91
Ø 2 (mm) hole (Section 4)	95	99
	98	99
	96	110
	95	89
	61	73
Aperture Ø 1 (mm) outlet of nozzle (Section 5)	98	89
	95	195
	56	180
	48	150
	55	180

**Table 3:** Ra of different focusing tube before polishing.

Inspected Items	Focusing Tube One Ra (μm)	Focusing Tube Two Ra (μm)	Judge OK or NG (μm)
Large inner hole	1.323	1.149	OK Ra ≤ 1.6
Small inner hole	0.472	0.519	OK Ra ≤ 1.6
Upper surface	0.745	0.869	OK Ra ≤ 1.6
lower surface	0.871	1.172	OK Ra ≤ 1.6

**Table 4:** Polishing technical parameters.

Parameters	Symbols	Unit	Value
Durability test	h	hour	480
Hydraulic pressure	p	Mpa	2.0
Mass flow rate of mixed liquid	v	L/min	10
Particle quality	-	Mesh (mm)	80 (0.177)
Outlet diameter of nozzle	do	mm	1
Outlet diameter of focusing tube	df	mm	10
Angle of cutting head	j	°	90
Particle diameter	-	1000 nm, 500 nm	Al_3_O_2_

**Table 5:** The percentage of metal contents in the wear area at the nozzle 1 and nozzle 2 exits.

Analysis Report of Figure 11c (Nozzle 1)							Analysis Report of Figure 12c (Nozzle 2)						
Elt.	Line	Intensity (c/s)	Atomic %	Atomic Ratio	Conc.	Units	Elt.	Line	Intensity (c/s)	Atomic %	Atomic Ratio	Conc.	Units
C	Ka	680.28	52.397	2.6527	42.241	wt.%	C	Ka	104.62	37.023	3.4390	17.184	wt.%
N	Ka	71.93	23.503	1.1899	22.096	wt.%	N	Ka	44.80	22.379	2.0788	12.113	wt.%
O	Ka	125.56	19.752	1.0000	21.211	wt.%	O	Ka	45.57	10.766	1.0000	6.656	wt.%
F	Ka	0.00	0.000	0.0000	0.000	wt.%	F	Ka	0.25	0.043	0.0040	0.032	wt.%
Al	Ka	5.02	0.082	0.0041	0.148	wt.%	Al	Ka	3.82	0.212	0.0197	0.221	wt.%
Si	Ka	12.89	0.188	0.0095	0.354	wt.%	Si	Ka	22.66	1.066	0.0991	1.157	wt.%
S	Ka	53.91	0.707	0.0358	1.522	wt.%	S	Ka	26.59	1.052	0.0977	1.303	wt.%
Ti	Ka	12.94	0.230	0.0117	0.739	wt.%	Ti	Ka	2.60	0.115	0.0107	0.213	wt.%
V	Ka	2.31	0.045	0.0023	0.155	wt.%	V	Ka	0.85	0.042	0.0039	0.083	wt.%
Cr	Ka	16.71	0.368	0.0186	1.284	wt.%	Cr	Ka	101.00	5.483	0.5093	11.017	wt.%
Fe	Ka	78.60	2.599	0.1316	9.743	wt.%	Fe	Ka	225.73	19.486	1.8101	42.054	wt.%
Ni	ka	2.54	0.128	0.0065	0.506	wt.%	Ni	Ka	13.16	1.768	0.1642	4.010	wt.%
Zn	ka	0.00	0.000	0.0000	0.000	wt.%	Zn	Ka	0.30	0.068	0.0063	0.172	wt.%
Total			100.000		100.000	wt.%				100.000		100.000	wt.%

**Table 6:** RMS of different nozzles after polishing.

Inspected Items	RMS After Polishing (nm)	
	Nozzle (1)	Nozzle (2)
Ø 9.5 (mm) hole (Section 1)	484	206
	481	200
	488	191
	480	192
	581	193
	493	177
	498	157
Ø 6 (mm) hole (Section 2)	470	185
	450	179
	491	177
	527	192
	591	193
	497	194
	490	192
Ø 3 (mm) hole (Section 3)	491	154
	484	168
	498	167
	528	199
	591	191
	490	197
	491	195
Ø 2 (mm) hole (Section 4)	485	191
	455	183
	487	185
	475	175
	508	183
Aperture Ø 1 (mm) outlet of nozzle (Section 5)	966	223
	1542	322
	1652	352
	1485	385
	1764	444

## Data Availability

The original contributions presented in this study are included in the article. Further inquiries can be directed to the corresponding author.

## Nomenclature

CNC SUN’5 Axis lathe	Computer numerical control
ZYGO	Laser scanning interferometer
AWJ	Abrasive water jet
Ra	Arithmetic mean deviation of the contour
BK7	Optical glass
RMS	Root mean square/Roughness Measurement of the Surface
